# The emerging role of miR-506 in cancer

**DOI:** 10.18632/oncotarget.11294

**Published:** 2016-08-15

**Authors:** Jian Li, Jingfang Ju, Bing Ni, Huaizhi Wang

**Affiliations:** ^1^ Institute of Hepatopancreatobiliary Surgery, Southwest Hospital, Third Military Medical University, Chongqing, PR China; ^2^ Translational Research Laboratory, Department of Pathology, Stony Brook University, Stony Brook, NY, USA; ^3^ Department of Pathophysiology and High Altitude Pathology, Third Military Medical University, Chongqing, PR China

**Keywords:** microRNAs, miR-506, cancer, tumor suppressor

## Abstract

MicroRNAs (miRNAs) are small non-coding RNAs that post-transcriptionally regulate gene expression. They are involved in almost all biological processes, and many have been identified as potential oncogenes or tumor suppressor genes. miR-506 was recently discovered to play pivotal roles in regulating cell proliferation, differentiation, migration and invasion. Dysregulation of miR-506 has been demonstrated in multiple types of cancers; however, whether it functions as an oncogene or a tumor suppressor seems to be context-dependent. Altered miR-506 expression in cancer is caused by promoter methylation and changes in upstream transcription factors. In this review, we summarize the current understanding of the diverse roles and underlying mechanisms of miR-506 and its involvement in cancer, and suggest the potential therapeutic strategy based on miR-506.

## INTRODUCTION

MicroRNAs (miRNAs) are a class of small (~22 nt), non-coding, single-stranded RNAs that regulate gene expression at the post-transcriptional level. miRNAs are generated from endogenously transcribed long primary transcripts (pri-miRNAs), which are further cleaved inside the nucleus by Drosha (RNase III), producing approximately 70-nucleotide stem-loop precursor miRNAs (pre-miRNAs) [1]. Pre-miRNAs are actively transported from the nucleus to the cytoplasm by Exportin 5 and are further processed by Dicer (RNase III) to yield mature miRNAs [2, 3]. The mature miRNAs are incorporated into RNA-induced silencing complex (RISC) and function as guides that promote binding to complementary sequence in the 3′-untranslated region (3′-UTR) of target genes, thereby triggering mRNA degradation or translational repression [4, 5]. It is estimated that miRNAs may regulate as much as 30% of the transcriptome [6]. The discovery of miRNAs has opened up a new field of post-transcriptional regulation of gene expression in organisms. Accumulating evidence suggests that dysregulation of miRNAs is involved in the pathogenesis of many types of human diseases, including cancers [7–9]. miRNAs can act as tumor suppressor genes or oncogenes in a wide range of human malignancies, involving multiple pathways and cellular functions in the development and progression of cancer [10–12].

**Figure 1:**
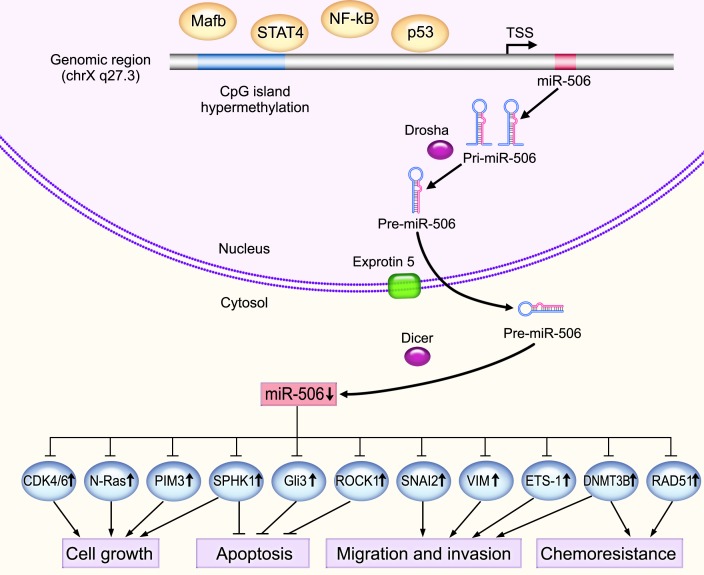
The underlying mechanisms of the tumor suppressive activity of miR-506 Mature miR-506 is processed by RNase III within and outside the nucleus by Drosha and Dicer, respectively. miR-506 is significantly downregulated in various types of cancers and functions as a tumor suppressor by targeting important oncogenes, such as N-Ras, PIM3, SPHK1, ROCK1 and ETS-1, thereby regulating important cancer-related processes, such as cell proliferation, apoptosis, senescence, chemoresistance, invasion and migration. DNA hypermethylation of the CpG islands in the promoter region of miR-506 blocks miR-506 transcription. In addition, several transcription factors can regulate miR-506 expression. For instance, NF-κB can bind to the upstream promoter region of miR-506 to suppress transcription.

miR-506 was first identified by Bentwich *et al*. [13] in the primate testis as a member of an X chromosome-linked miRNA cluster of ten miRNAs, which form a family of related sequences and generate seven distinct seeds (miR-506, miR-507, miR-508, miR-509, miR-510, miR-513, and miR-514). The entire miRNA cluster was found to be well conserved among primate species [14, 15]. Subsequently, Zhao *et al.* found that miR-506 acts as an anti-oncogenic miRNA in malignantly transformed human bronchial epithelial cells, and restoration of miR-506 in the transformed cells suppressed tumor growth *in vitro* and *in vivo* [16]. Another report demonstrated that miR-506 prevented TGF-β-induced epithelial-mesenchymal transition (EMT), augmented E-cadherin expression, and inhibited cell migration and invasion by targeting snail family zinc finger 2 (SNAI2). Decreased miR-506 expression in tumor tissues was significantly correlated with poor prognosis in ovarian cancer patients [17]. Changes in miR-506 expression have been subsequently discovered in other tumor types. However, the expression pattern and roles of miR-506 are complicated, even contradictory, in these reports, suggesting unique roles for miR-506 in different tumor types. Mounting evidence has demonstrated that miR-506 is a tumor suppressor gene [16–38] (Figure 1). However, in several cases, miR-506 appears to act as an oncogene [39, 40]. The purpose of this review is to highlight the emerging and diverse functions of miR-506 and its implication in cancer.

### Functions of miR-506 in cancer: evidence from both cultured cell lines and clinical samples

miRNAs orchestrate their functions primarily by binding to complementary sequences within the 3′-UTR of target mRNAs to diminish translation from these targets. Downregulation or upregulation of miR-506 has been shown to affect diverse biological behaviors by suppressing the translational output of several different target genes. Several miR-506 targets have known roles in various types of cancers (Table 1). Gene ontology (GO, http://www.geneontology.org/) analysis revealed that the targeted genes were related to diverse biological processes. Most of the targeted genes are downregulated in the specific diseases context and are associated with several physiological events including cell proliferation, cell differentiation, cell apoptosis, angiogenesis, cell migration and invasion (Table 1), suggesting the potential roles of miR-506 in normal cellular physiology. However, the exact physiological roles of miR-506 should be revealed in a knockout animal model in the future. In addition, miR-506 was also shown to be involved in the pathogenesis of primary biliary cirrhosis *via* targeting the Cl-/HCO3- Anion exchanger 2 mRNA [41] and regulating type III inositol 1,4,5-trisphosphate receptor (InsP3R3)-mediated Ca2+ signaling and secretion in cholangiocytes [42]. Here, we dissect the functions of miR-506 in different tumor types to highlight its diverse cellular functions.

**Table 1:** Verified direct targets of miR-506

GO analysis	Gene symbol	Description	Target gene expression	miR-506 expression	Diseases	References
Cell differentiation	CDH2	Cadherin 2, Type 1, N-Cadherin (Neuronal)	↑	↓	EOC	[32]
	STAT3	Signal Transducer and Activator Of Transcription 3	↑	↓	Glioma, neuroblastoma	[29,64]
	Gli3	GLI Family Zinc Finger 3	↑	↓	Cervical cancer	[33]
	FLOT1	Flotillin-1	↑	↓	ccRCC	[34]
	IQGAP1	IQ motif-containing GTPase activating protein 1	↑	↓	Breast cancer	[31]
						
Cell adhesion and migration	ITGB1	Integrin, Beta 1	↑	↓	Prostate cancer	[63]
	ITGB3	Integrin, Beta 3	↑	↓	Prostate cancer	[63]
	ROCK1	Rho-associated protein kinase 1	↑	↓	HCC	[25]
	CD151	CD151 Molecule (Raph Blood Group)	↑	↓	Breast cancer	[18]
	SNAI2	Snail family zinc finger 2	↑	↓	Ovarian cancer, breast cancer, osteosarcoma	[17,18,59]
	VIM	Vimentin	↑	↓	Ovarian cancer, Breast cancer	[18,32]
	ETS-1	V-Ets Avian Erythroblastosis Virus E26 Oncogene Homolog 1	↑	↓	GC	[28]
	E2H2	Enhancer of zeste homolog 2	↑	↓	Colon cancer	[37]
	SPON1	F-spondin 1	↑	↓	HCC	[22]
						
Response to PDGF	PDGFRB	Platelet-derived growth factor receptor beta	↑	↓	Glioblastoma	[72]
						
Blood vessel development	GATA6	GATA-binding protein 6	↑	↓	OSCC	[24]
	SPHK1	Sphingosine kinase 1	↑	↓	HCC, PC	[48,56]
						
Cell proliferation	CREB1	cAMP responsive element binding protein 1	↑	↓	Esophageal cancer	[35]
	PIM3	Pim-3 Proto-Oncogene, Serine/Threonine Kinase	↑	↓	PC	[26]
	CDK4	Cyclin-Dependent Kinase 4	↑	↓	Ovarian cancer, neuroblastoma	[19,64]
	CDK6	Cyclin-Dependent Kinase 6	↑	↓	Ovarian cancer	[19]
	N-Ras	Neuroblastoma RAS Viral (V-Ras) Oncogene Homolog	↑	↓	16HBE-T	[16]
	YAP	Yes-associated protein	↑	↓	HCC, GC, breast cancer	[20,23,27]
	FOXQ1	Forkhead box Q1	↑	↓	NPC, cervical cancer	[38,57]
	NF-κB p65	Nuclear Factor Of Kappa Light Polypeptide Gene Enhancer In B-Cells P65	↑	↑	Lung cancer	[61]
						
Response to ionizing radiation and drug	RAD51	RAD51 Recombinase, Double-strand DNA damage repair gene	↑	↓	Ovarian cancer	[45]
	DNMT3B	DNA methyltransferase 3B	↑	↓	CRC	[21]
	DNMT1	DNA methyltransferase 1	↑	↓	CRC	[21]
	PPARα	Peroxisome proliferator-activated receptor α	↓	↑	Colon cancer	[40]
	ABCC4	Multidrug resistance protein 4	↑	↓	HEK293T/17	[73]
						
Cell surface receptor signaling pathway	IGF2BP1	Insulin-Like Growth Factor 2 MRNA Binding Protein 1	↑	↓	Glioblastoma	[60]
	InsP3R3	Inositol1,4,5-trisphosphate receptor	↓	↑	Cholangiocyte	[42]
	AE2	Cl-/HCO3- Anion exchanger 2 gene	↓	↑	PBC	[41]

Abbreviation: EOC: epithelial ovarian cancer; HCC, Hepatocellular carcinoma; PC: pancreatic cancer; ccRCC, Clear cell renal cell carcinoma; OSCC, Oral squamous cell carcinoma; NPC, Nasopharyngeal carcinoma; CRC: colorectal cancer ; 16HBE-T : malignant transformation of human bronchial epithelial cells; HEK293T/17: Human embryonic kidney cells; PBC: Primary Biliary Cirrhosis.

## OVARIAN CANCER

Recent miRNA expression analyses revealed that a cluster of eight miRNAs, located on chrXq27.3, was down-regulated in patients with early-relapsing ovarian cancer, and miR-506 was among these miRNAs [43]. Ectopic expression of miR-506 inhibited cell proliferation and increased sensitivity to cisplatin in human ovarian cancer cellular models [43]. In another study, Yang *et al.* showed that miR-506 augmented E-cadherin expression, prevented TGF-β-induced EMT and inhibited cell migration and invasion by targeting SNAI2 in ovarian cancer [17]. The authors also demonstrated that miR-506 simultaneously suppressed Vimentin and N-cadherin, and silencing of Vimentin reversed EMT and inhibited cell migration and invasion in epithelial ovarian cancer (EOC) cells [32]. Thus, the authors concluded that miR-506 downregulation promotes an aggressive phenotype in ovarian carcinoma. In addition, the authors showed that miR-506 expression had an anti-proliferative effect on ovarian cancer cells. Overexpression of miR-506 inhibited proliferation and promoted senescence of ovarian cancer cells *via* direct targeting CDK4 and CDK6. miR-506 can suppress the CDK4/6-FOXM1 signaling pathway, which is activated in the majority of ovarian carcinomas [19]. Because the acquisition of EMT features has been associated with chemoresistance [44], the researchers also examined the role of miR-506 in chemotherapy response in high-grade ovarian cancers. miR-506 was associated with a better response to therapy and longer progression-free and overall survival, and miR-506 could augment the response to cisplatin and olaparib *via* targeting RAD51 to suppress homologous recombination-mediated repair of double-strand breaks in ovarian cancer cell lines [45, 46]. These findings confirm that miR-506 acts as a tumor suppressor in ovarian cancer.

miRNAs have been recognized as important prognostic biomarkers in disease management [47]. In the context of ovarian cancer, low levels of miR-506 were significantly associated with poor prognosis in three independent cohorts of ovarian cancer patients [17]. Another study confirmed that high miR-506 expression was positively correlated with early FIGO stage and longer survival in EOC, demonstrating that miR-506 can be used as a prognostic predictor for EOC patients [32]. Although a single miR-506 could be used as prognostic biomarker for ovarian cancer with considerable sensitivity and specificity, considering the innate heterogeneity of ovarian cancer, combination of a panel of relevant miRNAs including miR-506 may have more advantages and future studies are clearly needed to address this issue.

## PANCREATIC CANCER

We recently revealed that the miR-506 promoter is highly methylated in pancreatic cancer (PC) tissues and that reduced miR-506 expression was significantly associated with pathologic tumor status, distant metastasis, clinical stage, and decreased survival of PC patients. miR-506 suppressed cell proliferation, induced cell cycle arrest at the G1/S transition, and enhanced apoptosis and chemosensitivity of PC cells. In addition, we identified sphingosine kinase 1 (SPHK1) as a novel target of miR-506, the expression of which inhibited the SPHK1/Akt/NF-κB signaling pathway, which is activated in PC [48]. Our data suggest that miR-506 acts as a tumor suppressor miRNA and is epigenetically silenced in PC. Consistent with our study, Du *et al.* also found that the expression of miR-506 was significantly downregulated in PC tissues and negatively correlated with PIM3, a member of the proto-oncogene PIM family [26]. Further studies suggested that miR-506 acts as a tumor suppressor by suppressing PC cell proliferation, which was partially alleviated by PIM3 overexpression. Based on these data, it appears that miR-506 acts as a tumor suppressor in PC. However, as both studies are based on a relative small patient's specimen size, large scales of patient cohorts from multiple centers are needed to confirm the prognostic value of miR-506 in human PC.

## GASTRIC CANCER

To date, a large number of human endogenous miRNAs have been implicated in the pathological tumorigenesis and progression of gastric cancer (GC) [49–51]. Sakimura *et al.* found that the expression of miR-506 is downregulated in human GC and that low miR-506 expression is significantly associated with poor prognosis, poorly differentiated disease and high SNAI2 expression [30]. Further studies demonstrated that overexpression of miR-506 suppressed GC cell proliferation and migration *via* downregulating SNAI2, a transcriptional repressor of E-cadherin [30]. Deng *et al*. also demonstrated that miR-506 expression is decreased in GC patients and that low miR-506 expression is associated with tumor size, pathological tumor node metastasis stage, and lymph node metastases [23]. Ectopic expression of miR-506 inhibited cell proliferation, invasion, and EMT in GC cells by directly targeting Yes-associated protein 1 (YAP1). Meanwhile, reintroduction of Yap1 rescued the miR-506-induced effects on cell proliferation and invasion [23]. Another report confirmed that miR-506 is downregulated in metastatic gastric cancer cell lines and that low expression of miR-506 is associated with poor overall GC patient survival [28]. Further study suggested that miR-506 overexpression in GC inhibited endothelial cell angiogenesis and metastatic invasion *via* suppressing the proto-oncogene transcription factor ETS-1 [28], which plays an important role in angiogenesis and tumor metastasis [52]. Therefore, miR-506 functions as a tumor suppressor and has potential value for GC prognosis.

## BREAST CANCER

Many miRNAs, including miR-506, have demonstrated roles in the pathophysiology of breast cancer [53–55]. Arora *et al.* found that miR-506, predicted to target EMT-related genes, is significantly related to breast cancer patient survival [18]. Overexpression of miR-506 suppressed TGF-β-induced EMT and inhibited adhesion, invasion, and migration *via* downregulating mesenchymal genes such as SNAI2, Vimentin, and CD151. Further study suggested that NF-κB bound to the upstream promoter region of miR-506 to suppress transcription of the miRNA [18]. Yu *et al*. demonstrated that overexpression of miR-506 significantly suppressed the proliferation, colony formation, and migration of breast cancer cells [36]. Another study demonstrated that miR-506 was downregulated in human breast cancer tissues and cell lines and that the expression level of miR-506 was reduced with increasing tumor stage [31]. Gain-of-function and loss-of-function experiments revealed that overexpression of miR-506 inhibited cell proliferation, adhesion and invasion by directly targeting the IQ motif-containing GTPase activating protein 1 (IQGAP1) and repressing the downstream extracellular signal regulated kinase (ERK) mitogen-activated protein kinase (MAPK) signaling pathways. In addition, IQGAP1 rescued the suppressive effect of miR-506 on cell proliferation, adhesion, invasion, and the activation of ERK/MAPK signaling [31]. Hua *et al.* confirmed that miR-506 was commonly downregulated in breast cancer and that miR-506 suppressed cellular proliferation, migration and invasion by directly binding to the 3′-UTR of YAP mRNA [27]. These reports provide evidence that miR-506 is frequently downregulated in breast cancer and acts as a tumor suppressor gene. Nevertheless, there are still limited studies to investigate the potential role of miR-506 as biomarker for diagnosis and/or follow-up of breast cancer, which has to be clarified in the future.

## HEPATOCELLULAR CARCINOMA

Wang *et al.* demonstrated that the expression of miR-506 was significantly lower in hepatocellular carcinoma (HCC) tissues and negatively correlated with YAP [20]. A further study suggested that miR-506 significantly inhibited the proliferation of HepG2 and H7402 cells *via* targeting YAP and repressing the YAP-targeting genes c-Myc and connective tissue growth factor (CTGF). In another study, the expression level of miR-506 was negatively correlated with SPHK1 in HCC, and miR-506 suppressed angiogenesis in HCC by targeting SPHK1 [56]. This is the first reported study that highlighted the pivotal role of miR-506 in tumor angiogenesis, basing on human umbilical vein endothelial cell (HUVEC) tube formation assay *in vitro*. However, miR-506 knockdown or knockout animal models is warranted to elucidate the exact roles of miR-506 on tumor angiogenesis in the future. Deng *et al.* also found that miR-506 significantly inhibited HCC cell proliferation *in vitro* and tumorigenicity *in vivo* and identified Rho-associated protein kinase 1(ROCK1) as a novel target of miR-506 [25]. Dai *et al*. confirmed that miR-506 is downregulated in HCC and inhibits proliferation, migration and invasion by suppressing F-spondin 1 (SPON1). Restoring SPON1expression and silencing of SPON1 *in vitro* reversed the effects of miR-506 mimics and inhibitors, respectively [22]. Therefore, all these findings highlight the tumor suppressive roles of miR-506 in HCC.

## COLORECTAL CANCER

Zhang *et al.* reported that miR-506 was downregulated in colon cancer cell lines and tumor tissues, and miR-506 expression was inversely correlated with tumor size, lymph node invasion, TNM stage, and metastasis. Furthermore, low levels of miR-506 were associated with poor prognosis [37]. miR-506 inhibited the proliferation and metastasis of colon cancer *via* binding to the 3′-UTR of enhancer of zeste homolog 2 (EZH2), a member of the Polycomb group (PcG) protein family. Restoration of EZH2 expression partially alleviated the effects of miR-506-overexpressing colon cancer cells. Moreover, the miR-506-EZH2 axis suppressed proliferation and metastasis by activating/inhibiting specific downstream tumor-associated genes and the Wnt/β-catenin signaling pathway [37]. In another study, Chen *et al.* elucidated that miR-506 and miR-124 levels were significantly reduced in human colorectal cancer (CRC) tissues and that miR-506 and miR-124 inhibited progression and enhanced sensitivity to chemotherapy by downregulating DNMT3B and DNMT1 in CRC [21]. In addition, Tong *et al.* reported that miR-506 was overexpressed in hydroxycamptothecin (HCPT)-resistant human colon cancer cells and that miR-506 conferred HCPT resistance by targeting PPARα [40]. These studies suggest that miR-506 could either inhibit or enhance chemoresistance of CRC cells in a cell-type-dependent manner. Further clinical investigation and animal experiment are needed to dissect the paradoxical functions of miR-506 in different genetic context.

## OTHER TUMOR TYPES

In addition to the aforementioned tumors, the tumor suppressive role of miR-506 has been widely investigated in many other malignancies, including esophageal cancer [35], glioma [29], oral squamous cell carcinoma [24], cervical cancer [33, 57], clear cell renal cell carcinoma [34, 58], nasopharyngeal carcinoma [38], osteosarcoma [59], glioblastoma [60], and lung cancer [61] (Table 2). Moreover, other studies have investigated the potential roles of miR-506 in cancer. Through subpathway analysis of each subtype of head and neck squamous cell carcinoma (HNSCC), An *et al.* demonstrated that several miRNAs (miR-506, let-7a, miR-1, miR-206, miR-153, and miR-519a) and their target genes play crucial roles in the prevention of HNSCC *via* regulating several distinct pathways [62]. Li *et al.* reported that miR-506 was the most significant miRNA screened by a bioinformatics strategy in prostate cancer and was found to regulate genes including ITGB1 and ITGB3 by binding the target sequence GUGCCUU [63]. Therefore, miR-506 might play an important role in the pathogenesis of prostate cancer [63]. Using a high-content morphological screen to identify differentiation-inducing miRNAs in neuroblastoma, Zhao *et al.* found that the most potent inducer of differentiation was the miR-506-3p/miR-124-3p seed family and that miR-506-3p expression but not miR-124-3p is dramatically upregulated in differentiated neuroblastoma cells, indicating an important role for endogenous miR-506-3p in differentiation and tumorigenesis [64]. Additional investigations are certainly warranted to fully characterize the function of miR-506 in these cancers.

Despite the well characterized role of miR-506 as a tumor suppressor, emerging evidence indicates that miR-506 functions as an oncogene in melanoma and confers chemoresistance in colon cancer. Streicher *et al*. reported that miR-506, a member of the miR-506-514 cluster, was consistently overexpressed in almost all melanoma samples and suggested it had an oncogenic role, initiating melanocyte transformation and promoting melanoma growth [39]. Clearly, melanoma is a cancer that is quite different and unique from other tumor types and it response well to immunotherapy while other solid tumor types do not. DNA sequence analysis revealed that the upstream of the miR-506-514 cluster did not have strong binding sites for transcription factors relevant to cancer, indicating a novel mechanism for upregulation of the miRNA cluster [39]. The gene expression changes caused during melanocyte transformation might provide a clue. However, the miR-506-514 cluster is upstream of several pathways involved in tumor development and progression, further work will be needed to understand whether expression of this cluster is regulated by transcription factors correlated with these pathways [39]. In addition, using miRNA microarray analyses, Tong *et al*. demonstrated that miR-506 was overexpressed in hydroxycamptothecin (HCPT)-resistant human colon cancer cells and that miR-506 conferred cancer cells resistance to HCPT by inhibiting PPARα expression [40].

**Table 2:** Summary of representative studies investigating the role of miR-506 in clinical samples

Cancer types	miR-506 expression	miR-506 function	Prognosis of low miR-506	References
Ovarian cancer	↓	TSG	Poor	[17,32,45]
HCC	↓	TSG	Poor	[20,56]
Breast cancer	↓	TSG	Poor	[18]
Gastric cancer	↓	TSG	Poor	[23,28,30]
Colon cancer	↓	TSG	Poor	[37]
ccRCC	↓	TSG	Poor	[34]
Pancreatic cancer	↓	TSG	Poor	[48]
Cervical cancer	↓	TSG	UD	[33]
OSCC	↓	TSG	UD	[24]
Esophageal cancer	↓	TSG	UD	[35]
Glioma	↓	TSG	UD	[29]
NPC	↓	TSG	UD	[38]
Lung cancer	↑	TSG	Poor	[61]
Melanoma	↑	Oncogene	UD	[39]

Abbreviation: HCC, Hepatocellular carcinoma; ccRCC, Clear cell renal cell carcinoma; OSCC, Oral squamous cell carcinoma; NPC, Nasopharyngeal carcinoma; TSG, Tumor suppressor gene; UD, undetermined.

## REGULATION OF MIR-506 EXPRESSION

Similar to protein-encoding genes, different regulatory mechanisms can control miRNA expression at a genetic or epigenetic level, and miRNA expression can also be affected by the dysregulation of specific transcription factors [65]. Recent studies have demonstrated that epigenetic inactivation is a common method of silencing miRNAs [66, 67]. Yang *et al.* identified five CpG sites in the promoter region of the miR-506 gene in ovarian cancer. Using quantitative pyrosequencing following sodium bisulfite treatment of DNA isolated from ovarian cancer tissues, the authors demonstrated that two of the five methylation sites exhibited trends of negative correlation between methylation and miR-506 expression. Treating the ovarian cancer cell line SKOV3 with 5-aza-2′-deoxycytidine (5-Aza-dC), a demethylating agent, significantly restored miR-506 levels [17]. Our group compared miR-506 promoter methylation levels between PC tissues, adjacent non-cancerous tissues and normal pancreatic tissues and found that DNA hypermethylation may account for the downregulation of miR-506 in PC. Treating PC cell lines with 5-Aza-dC resulted in a significant upregulation of miR-506 [48]. Meanwhile, Arora *et al*. revealed a putative NF-κB binding site at −1013 bp from precursor miR-506 through promoter sequence analysis. The authors confirmed that NF-κB bound to the upstream promoter region of the miR-506 gene to suppress transcription by chromatin immunoprecipitation (ChIP) assays, and miR-506 expression was induced by the suppression of NF-κB in breast cancer cell lines [18]. In addition, Zhao *et al.* demonstrated that transcription factors Mafb and STAT4 negatively regulate miR-506, indicating another regulatory network that could regulate miR-506 expression [68]. With respect to the upregulation of miR-506, Yin *et al.* found a putative p53-response element approximately 782 bp upstream of miR-506, and qPCR results revealed a significant correlation between miR-506 and p53 mRNAs levels in stage I lung cancer patients. miR-506 was markedly induced by the adriamycin treatment in lung cancer cell lines that express functional p53 [61] (Figure 1).

## THERAPEUTIC POTENTIAL OF MIR-506

Based on the critical roles of miRNAs in cancer, miRNAs exhibit great potential as novel therapeutic agents. A growing body of evidence has revealed that miR-506 is one of the most significantly downregulated miRNAs in various types of cancer and plays important tumor suppressive roles *in vitro* and *in vivo* [22, 33, 37]. Yang *et al.* reported that delivery of miR-506 incorporated in DOPC nanoliposomes in orthotopic ovarian cancer mouse models inhibited tumor growth and led to E-cadherin expression [17]. In another study, systemic delivery of miR-506 significantly enhanced the effect of cisplatin and olaparib in orthotopic ovarian cancer models [45]. Our previous study demonstrated that lentivirus-mediated miR-506 overexpression could inhibit PI3K/Akt signaling, impair the viability of PC cells, and slow the growth of PC xenografts in mice [48]. Moreover, miR-506 has been shown to inhibit the proliferation and tumorigenicity of several types of cancer cells [22, 25, 29, 33, 37] and suppress migration *in vitro* and *in vivo* [21, 37]. Thus, these results provide a strong rationale for utilizing miR-506 analogues to treat cancer in the future. However, we must bear in mind that the function of miR-506 is cell-specific, and a thorough understanding of its diverse functions is crucial for developing miR-506-based therapies. With miR-34 currently in a Phase I clinical trials as the first miRNA mimic for cancer treatment, all eyes are on the progress of miRNA-based therapeutics [69]. Although many hurdles may lie ahead, such as the low stability of synthetic RNA *in vivo*, effective delivery of miRNA and retention of miRNAs [70, 71], we are optimistic about the future of miRNA-based cancer therapeutics.

## CONCLUSIONS AND PERSPECTIVES

Most of the reported studies clearly demonstrated that miR-506 is commonly downregulated and acts primarily as a tumor suppressor in various cancers (Table 2). Evidence from both clinical samples and cancer cell lines suggest that dysregulation of miR-506 plays crucial roles in cancer development. However, considering the limited numbers of studies available and the bidirectional and context-specific roles of miR-506 (e.g., in melanoma), more investigations are warranted because melanoma is a unique disease in several aspects. The following molecular and cellular mechanistic questions may deserve future investigations. The normal physiologic role of miR-506 needs to be fully elucidated in a knockout or knockdown animal model. Secondly, the upstream molecular regulators of miR-506, which are responsible for aberrant expression of miR-506, such as NF-κB [18], p53 [61], Mafb and STAT4 [68], need to be explored. Additional targets of miR-506 need to be identified to reveal additional pathways to TGF-β [17], Akt [48], NF-κB [61], Wnt/β-catenin [37], STAT3 [29], Gli3 [33], and FOXM1 [19] that are regulated by miR-506. These studies would help us to better understand the pleiotropic functions of miR-506 in cancer development. Last, additional studies are clearly needed to fully validate the usefulness of miR-506 as a diagnostic and/or prognostic biomarker.
